# Case Report: Overcoming calcified nodules by repeated aggressive debulking and stent-free percunatenous coronary intervention using a drug-coated balloon

**DOI:** 10.3389/fcvm.2025.1674711

**Published:** 2025-11-10

**Authors:** Atsushi Funatsu, Kenshi Ono, Ryotaro Tani, Hideki Maeda, Masahiro Mizobuchi, Satoshi Akabame, Tomoko Kobayashi, Norihito Nakamura, Sho Torii, Gaku Nakazawa, Shigeru Nakamura

**Affiliations:** 1 Cardiovascular Center, Kyoto Katsura Hospital, Kyoto, Japan; 2 Department of Cardiology, Tokai University School of Medicine, Isehara, Kanagawa, Japan; 3 Department of Cardiology, Kindai University, Osaka, Japan

**Keywords:** calcified nodules, debulking, drug coated balloon (DCB), restenosis, stent-less intervention

## Abstract

**Background:**

The outcomes of percutaneous coronary intervention (PCI) for calcified nodules (CNs) has been unsatisfactory results, even with the use of drug eluting stents. The optimal treatment strategy for CNs continues to be a matter of debate, with no definitive solution in sight.

**Case Presentation:**

We present a case of a 76-year-old male with recurrent restenosis due to a CNs in the ostium of left circumflex artery. He underwent repeated stent-free PCI with aggressive debulking using atherectomy devices followed by drug coated balloon (DCB).

**Outcome:**

The patient experienced recurrent restenosis every six months; however, after undergoing three sessions of stent-free PCI using atherectomy device and DCB, the lesion remained free of restenosis witch was confirmed at 4 years follow up.

**Conclusion:**

This case highlights the potential of repeated stent-free PCI with debulking of calcified tissue followed by DCB angioplasty as a viable treatment option for CNs.

## Introduction

Even in the modern era where percutaneous coronary intervention (PCI) technologies and techniques have matured, calcified coronary lesions remain a challenging entity. These lesions are associated with poor device deliverability and limited lesion expandability, resulting in lower initial procedural success rates compared to non-calcified lesions (1). Furthermore, even with the use of contemporary drug-eluting stents (DES), long-term outcomes remain inferior in comparison to non-calcified lesions (2).

Among calcified lesions, calcified nodules (CNs) are considered a particularly unique and complex subtype. Their pathological characteristics are still not fully understood, and several studies have shown that CNs are associated with a higher risk of target vessel revascularization and stent thrombosis following PCI (3–6).

Calcified nodules are classified into two morphological types: eruptive and non-eruptive (7). Although eruptive nodules generally allow for better stent expansion compared to non-eruptive ones, both types are associated with a high incidence of target lesion failure (TLF), even when treated with contemporary DES. As for drug coated balloons (DCB), we often use them for treating CNs in our clinical practice to avoid permanent metal implantation. However, according to our data, the rate of target lesion revascularization (TLR) in CNs is 30% at 1.5 years, which is higher than that observed in sheet-like calcification (8). Moreover, repeat DCB after DCB failure is an independent predictor of TLR.

In the current situation, no matter what treatment is done, the outcomes for CNs lesions remain poor. What we can do now is to provide tailored treatment for each case, using the most optimal devices available, while leaving options for future interventions.

Recently, we experienced a very impressive case that could provide helpful hints for overcoming CNs.

## Case description

**Figure 1:**
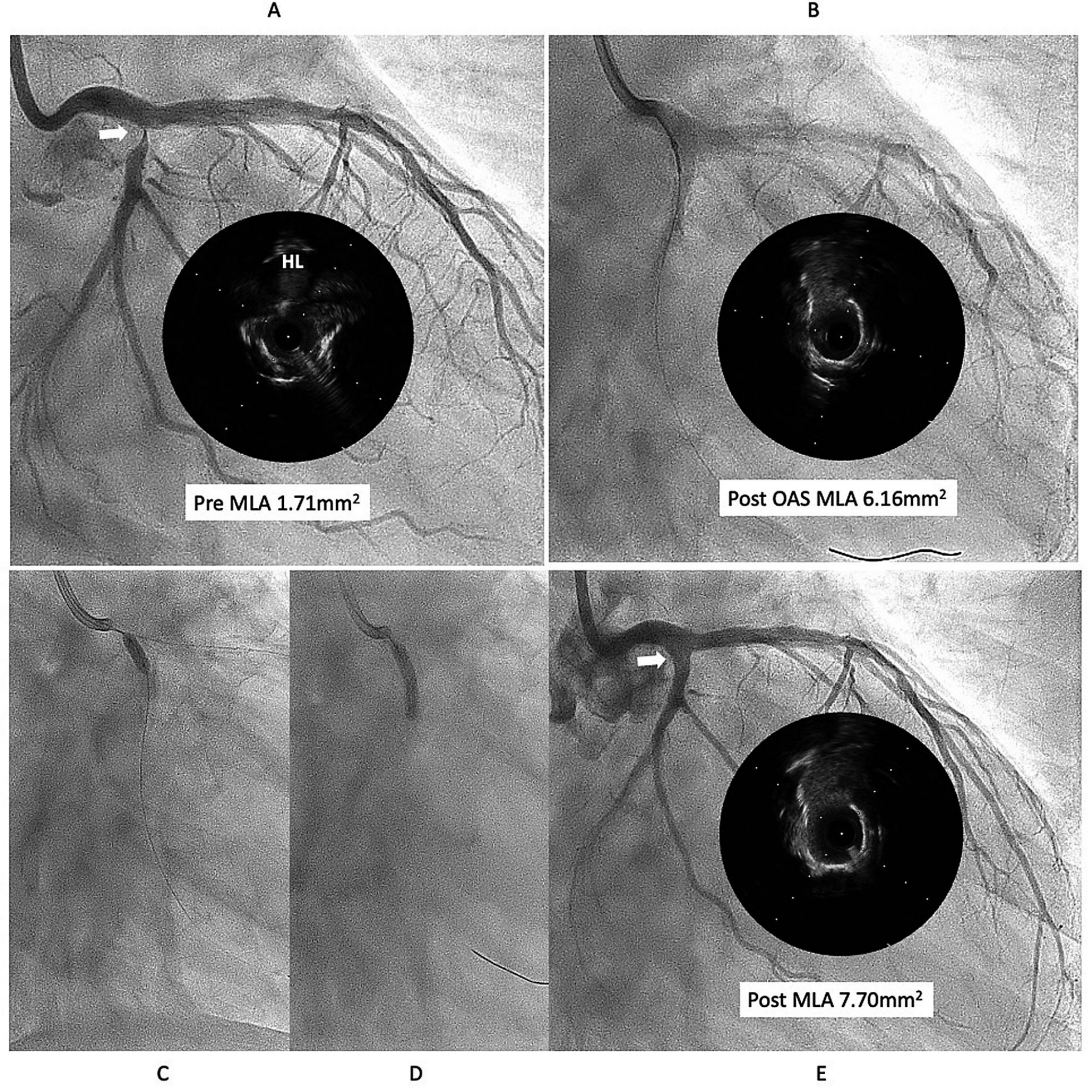
First intervention for left circumflex (LCX) ostial lesion (white arrow). **(A)** The intravascular ultrasound (IVUS) showed that calcified plaque is protruding into the lumen and surface is relatively smooth, that indicates the lesion is a non-eruptive calcified nodule (CN). **(B)** Diamondback micro crown was used to reduce the calcified tissue, with 7 runs at low speed and 6 runs at high speed. IVUS after ablation demonstrated a significant cutting effect, and lumen was well enlarged. **(C,D)** lesion was dilated using Wolverine cutting balloon 3.0 × 10 mm, followed by SeQuent Please 3.5 × 20 mm. **(E)** Final angiogram and IVUS showed good lesion dilation (white arrow). HL indicates high-lateral branch. MLA indicates minimum lumen diameter. OAS indicates orbital atherectomy system.

This case is a 76year-old male with chronic renal failure (CRF) on hemodialysis (HD). He admitted to our hospital due to chest pain during HD. Coronary angiography (CAG) revealed a very eccentric, calcified lesion in the ostium of the left circumflex artery (LCX) (Figure 1A), so we decided to perform PCI for this lesion. Intravascular ultrasound (IVUS) showed that the calcified plaque was protruding into the lumen, with a relatively smooth surface, indicating a non-eruptive calcified nodule (CN). The wire bias was on the plaque side, suggesting that the use of atherectomy devices would be safe and effective. Therefore, we performed debulking treatment using an orbital atherectomy system (OAS). The Diamondback 360 Micro Crown was used for the procedure at low and high rotational speed. Post-OAS IVUS demonstrated a significant cutting effect, and the lumen was enlarged (Figure 1B). The lesion was then dilated using a Wolverine cutting balloon (CB) 3.0 × 10 mm, followed by a SeQuent Please DCB 3.5 × 20 mm. The final angiogram showed no residual stenosis (Figures 1C–E).

**Figure 2:**
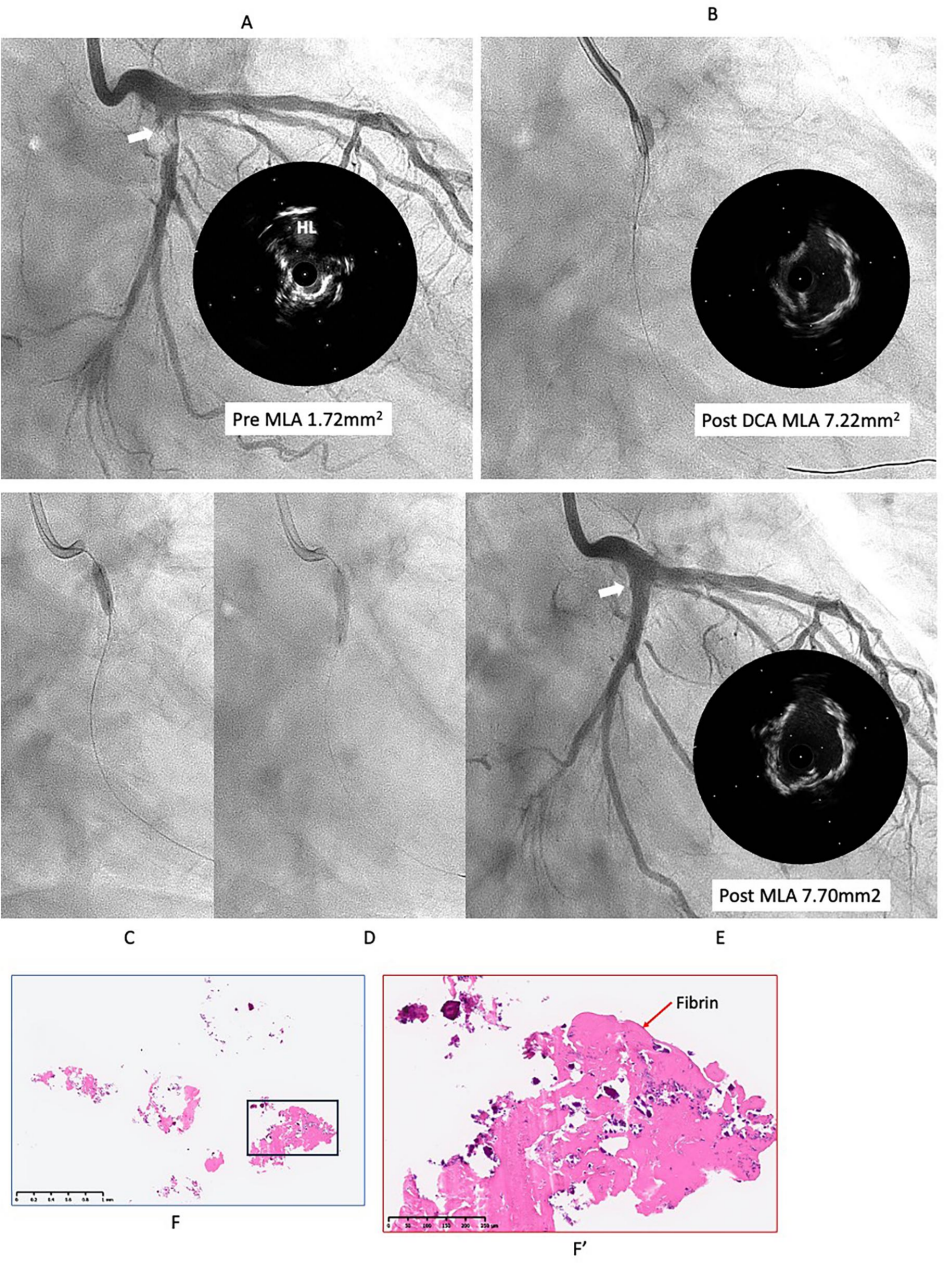
Second intervention for LCX ostial lesion (white arrow). **(A)** Angiography and IVUS showed restenosis with irregular surface. Lumen was filled by proliferative calcified tissue that was like non-eruptive CN. **(B)** Directional coronary atherectomy (DCA) was performed to reduce the CN using Atherocut (NIPRO), and then lumen was much gained. **(C,D)** The lesion was dilated using Wolverine cutting balloon 3.25 × 10 mm, which is one-quarter size up from previous one, followed by SeQuent Please 3.5 × 20 mm. **(E)** Final angiogram and IVUS showed good result with more circular lumen compared to the first intervention (white arrow). Low- **(F)** and high- (F′) power images of the tissue collected through directional coronary atherectomy revealed the calcified nodule with fibrin deposition (red arrow). Abbreviations are as defined in Figure 1.

**Figure 3:**
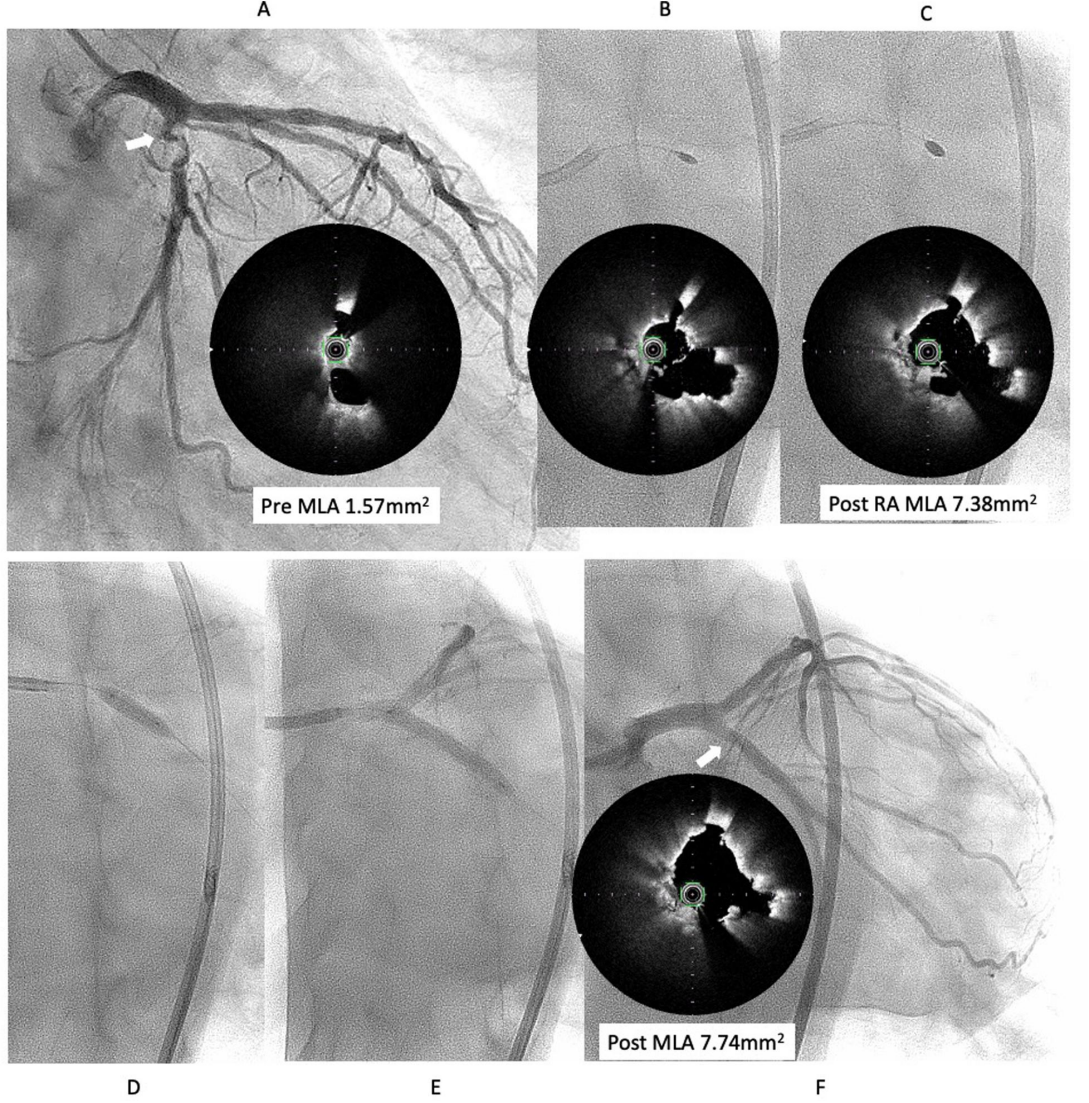
Third intervention for LCX ostial lesion (white arrow). **(A)** Angiography showed restenosis with irregular channel. Optical frequency domain imaging (OFDI) (TERUMO) showed very eccentric lumen with an irregular surface. Eruptive and non-eruptive CNs were present together. **(B,C)** Rotablator (Boston Scientific) was performed using 1.75 mm and 2.25 mm largest burr. **(D,E)** The lesion was dilated using a Wolvarine 3.75 mm, which is one-quarter size up from previous PCI, followed by a SeQuent Please 3.5 × 25 mm **(F)** Final angiogram and ODFI showed good lumen gain (white arrow). Abbreviations are as defined in Figure 1.

However, six months later, the symptoms recurred, and CAG revealed restenosis at the LCX (Figure 2A). Therefore, we proceeded with repeat PCI. The lesion appeared hazy on angiography, with small channels visible within the lesion. We selected an 8-French system, considering the possible need for a large-diameter Rotablator. IVUS showed that the lumen was filled with proliferative calcified tissue. While IVUS indicated features suggestive of a CN, it had already been treated six months earlier, so the proliferative CN tissue was assumed to be not very hard. Therefore, we attempted to remove the CN using directional coronary atherectomy (DCA). We selected a medium-size ATHEROCUT catheter (NIPRO, Japan) and performed a total of 31 cuts at a maximum pressure of 40 psi. After removing a total of 12.2 mg of CN tissue with DCA, the lumen was significantly enlarged (Figure 2B). Next, the lesion was dilated using a CB 3.25 mm, which was a quarter size larger than the previous one, followed by a SeQuent Please 3.5 × 20 mm (Figures 2C,D). The final angiogram showed a no residual stenosis without any complications (Figure 2E). However, 6 months later, the symptoms recurred, and CAG showed restenosis again with irregular channel (Figure 3A). Despite recurrent restenosis of the lesion, we decided to perform repeat PCI for this lesion because the patient was at high risk for bypass surgery due to hemodialysis. This time, we also chose an 8Fr system. We needed to take a different approach from the previous two procedures. OFDI revealed a very eccentric lumen with an irregular surface. Both eruptive and non-eruptive CNs were present in the same lesion.

**Figure 4:**
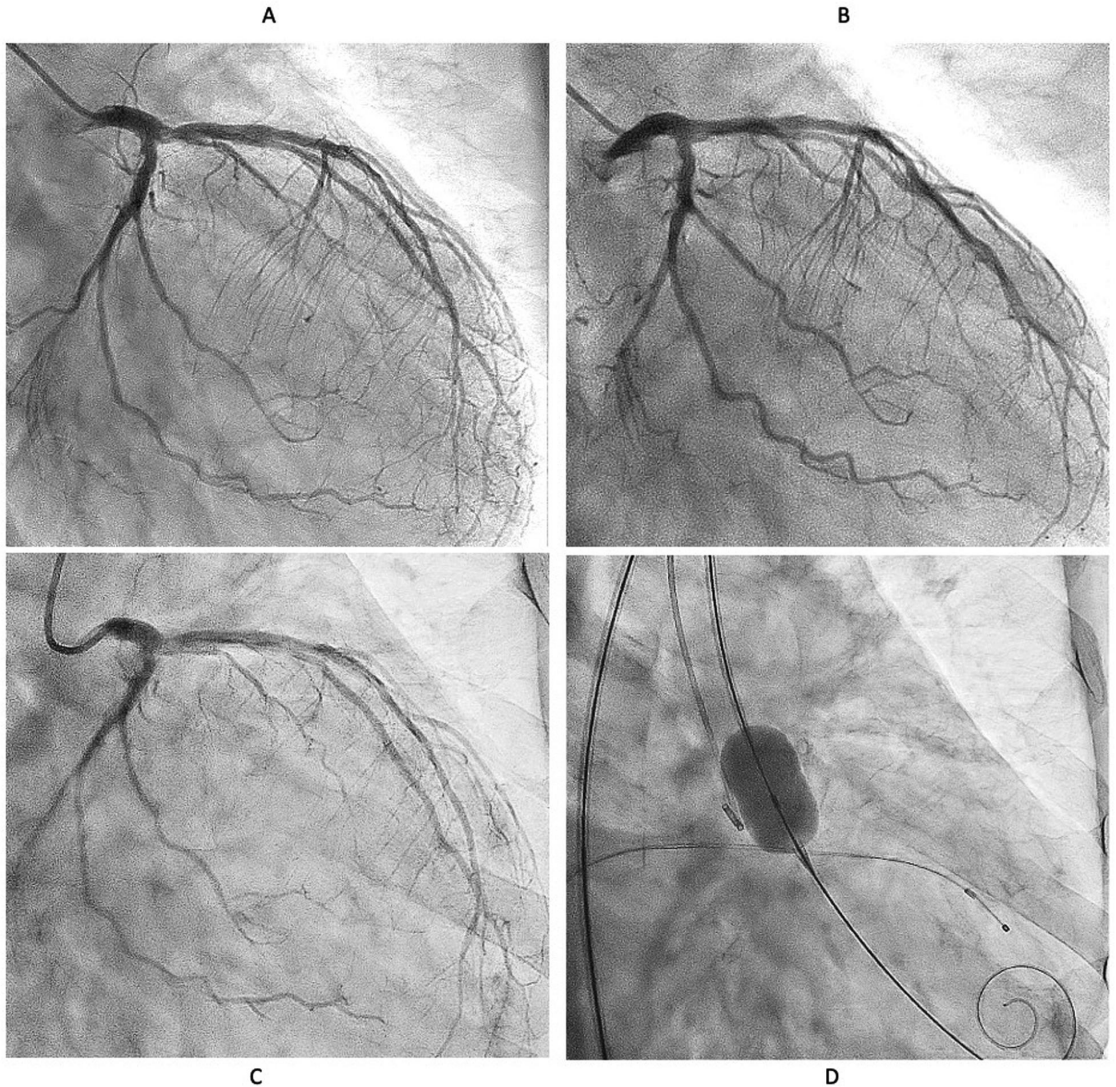
Serial angiographical follow up. **(A)** Angiography at 6 months showed no restenosis. Left anterior descending (LAD) ostial lesion was treated Rotablator followed by DCB. **(B)** No restenosis was observed at one year both CX and LAD. **(C)** Angiography revealed no restenosis even after 4 years. **(D)** Severe Aortic valve stenosis was treated with balloon aortic valvuloplasty instead of transcatheter aortic valve implantation because the patient was in the terminal stage of colon cancer.

We decided to perform rotational atherectomy using a large burr. First, a 1.75 mm burr was used. OFDI showed an enlarged lumen surrounded by residual CN. Subsequently, a 2.25 mm burr was then used for further debulking. A repeat OFDI then demonstrated further lumen enlargement without evidence of vessel wall injury (Figures 3B,C). Then, the lesion was dilated using a CB 3.75 × 10 mm, which is a quarter size up from previous PCI, followed by a SeQuent Please 3.5 × 25 mm. Final angiogram showed no residual stenosis without any complication (Figures 3D–F). Six months later, the ostium of the left anterior descending artery (LAD) was treated, with no restenosis the observed in the CX (Figure 4A). At one-year follow-up, there was no restenosis in either the CX and LAD ostium (Figure 4B). Four years later, the complained of chest pain again, so CAG was performed. However, the CAG revealed no restenosis at CX, even after 4 years (Figure 4C). He had severe aortic valve stenosis (AS) but was in the terminal stage of colon cancer. Therefore, we performed balloon aortic valvuloplasty for the severe AS (Figure 4D). We ultimately succeeded in overcoming the CNs in this patient.

## Discussion

Torii et al. reported that CNs protrudes into the lumen due to the fragmentation of calcified tissue at the hinge point (9). In our case, we hypothesize that the repeated recurrence of CNs eventually ceased because the overall volume of calcified tissue was finite and gradually reduced with each intervention. Initially, mechanical stress and interventions may have caused fragments of calcified tissue to protrude into the lumen, leading to restenosis. However, through repeating revascularization aimed at removing CNs, the reservoir of calcified tissue may have progressively diminished (Supplementary Figure S1). As the amount of residual calcified tissue decreased, the extent of new CNs protrusion also appeared to decrease, which could explain why restenosis eventually stopped. In other words, the pathophysiological mechanism behind the recurring protrusion may have been gradually exhausted due to the limited supply of calcific burden in the vessel wall. Of course, this hypothesis assumes that there is no significant new deposition of calcium over time. Should new calcified tissue develop over a long course—due to ongoing atherosclerotic processes—there is a possibility that restenosis may recur. However, considering the slow progression of vascular calcification in general, it is plausible that such a recurrence, if it happens, would require several years to become clinically significant (10).

In this case, three different atherectomy devices were used in each of the three PCI procedures. This choice was primarily left to the operator's discretion and does not carry significant meaning. The prevention of restenosis is thought to be primarily due to the repeated debulking of CNs, rather than the use of the Rotablator during the third intervention, as no significant differences in the post-procedural minimun lumen area were observed among the three treatments.

Currently, the standard treatment for calcified lesions involves the implantation of a DES following adequate debulking of calcified tissue. However, CN often protrude into the stent lumen, potentially compromising long-term outcomes.

In recent years, intravascular lithotripsy (IVL) has gained attention as a novel device for the treating calcified lesions. Ali et al. reported in a pooled analysis of the Disrupt CAD study that pre-dilation with IVL prior to DES implantation enabled adequate stent expansion even in CN lesions, comparable to that achieved in non-CN lesions, with similar long-term outcomes (11).

However, several unfavorable factors were present in this case beyond the presence of a CN. These included a lesion involving the LCX ostium, which is a known predictor of in-stent restenosis (12), and the fact that the patient is on dialysis (4). Given these high-risk features, the long-term outcome after DES implantation would likely be poorer than those reported in clinical studies. Therefore, we determined that DES implantation should be avoided in this case.

The therapeutic efficacy of drug-coated balloons (DCBs) for CN lesions remains insufficiently supported by data, and the topic has not yet been thoroughly discussed. However, even within CN lesions, non-calcified plaque and segments of relatively healthy vessel wall are often present. In such areas, the antiproliferative effect of the DCB can be expected, and it is considered that DCBs may help suppress restenosis more effectively than plain balloon angioplasty. Accordingly, it is considered that the DCB played a partial role in suppressing restenosis in this lesion.

## Conclusions

We experienced a CN case in which repeated stent-free treatment using a 3 different atherectomy device, and a drug-coated balloon successfully prevented restenosis. Reducing the volume of calcified plaque at the lesion using debulking devices may be a key strategy for achieving successful DCB outcomes in complex coronary artery CN lesions.

## Data Availability

The original contributions presented in the study are included in the article/Supplementary Material, further inquiries can be directed to the corresponding author.

## Supplementary material

Supplementary Figure S1 Our hypothesis for the overcoming of calcified nodules (CNs). Although a substantial portion of the protruding CN (gray area) can be excised during the initial atherectomy, recurrent CN protrusion into the vascular lumen is frequently observed within several months. Notably, the calcified tissue (blue area) underlying the CN gradually decreases over time. With repeating revascularizations for recurrent CN protrusion, serial debulking procedures result in progressive reduction of the underlying calcified tissue. Consequently, the extent of CN protrusion into the lumen diminishes, ultimately disrupting the cycle of restenosis driven by recurrent CN formation.
