# Supplementary figures and images for: Case Report: Overcoming calcified nodules by repeated aggressive debulking and stent-free percunatenous coronary intervention using a drug-coated balloon
Source: Front Cardiovasc Med. 2025 Nov 10;12:1674711. doi: 10.3389/fcvm.2025.1674711 (PMC12640911; doi:10.3389/fcvm.2025.1674711)

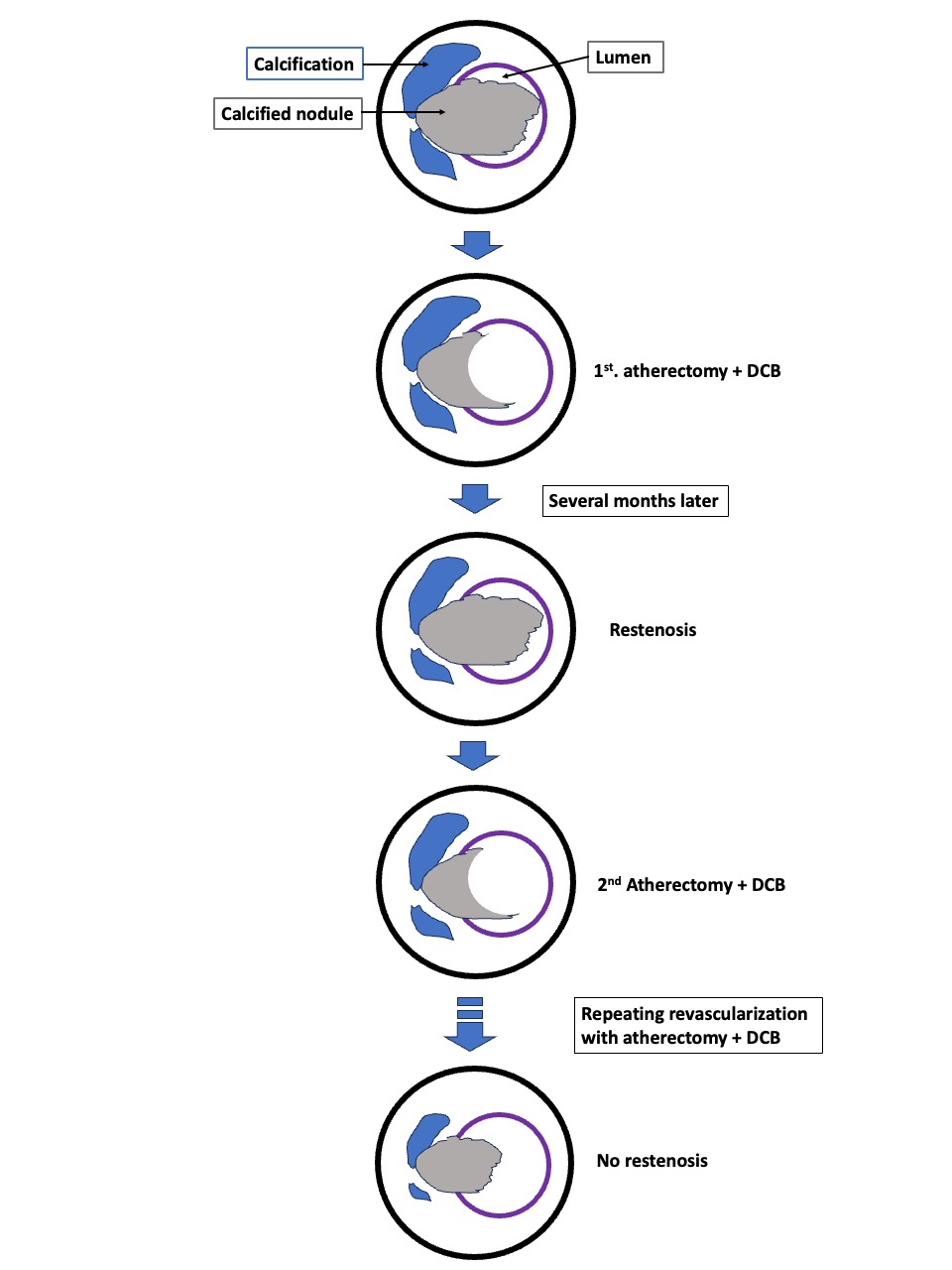

Supplement: Supplementary Figure S1 — Our hypothesis for the overcoming of calcified nodules (CNs). Although a substantial portion of the protruding CN (gray area) can be excised during the initial atherectomy, recurrent CN protrusion into the vascular lumen is frequently observed within several months. Notably, the calcified tissue (blue area) underlying the CN gradually decreases over time. With repeating revascularizations for recurrent CN protrusion, serial debulking procedures result in progressive reduction of the underlying calcified tissue. Consequently, the extent of CN protrusion into the lumen diminishes, ultimately disrupting the cycle of restenosis driven by recurrent CN formation. [file Image1.jpeg]
